# MiR-203-3p inhibits the oxidative stress, inflammatory responses and apoptosis of mice podocytes induced by high glucose through regulating Sema3A expression

**DOI:** 10.1515/biol-2020-0088

**Published:** 2020-12-22

**Authors:** Jingfu Chen, Qing Xu, Wei Zhang, YuLan Zhen, Fei Cheng, Guo Hua, Jun Lan, Chang Tu

**Affiliations:** Department of Cardiovascular Medicine and Dongguan Cardiovascular Institute, The Third People’s Hospital of Dongguan City, No. 1, Xianglong Road, Shi Long Town, Dongguan, China; Department of Cardiology, Huangpu Division of The First Affiliated Hospital, Sun Yat-sen University, Guangzhou, China; Department of Oncology, The Third People’s Hospital of Dongguan City, Dongguan, China

**Keywords:** diabetic nephropathy, miR-203-3p, Sema3A, oxidative stress, inflammatory responses, apoptosis

## Abstract

Diabetic nephropathy (DN) is the most serious long-term microvascular complication of diabetes, which mainly causes podocyte injury. Many studies have shown that microRNAs play a vital role in the development of DN. Studies have shown that miR-203-3p is involved in mesangial cell proliferation and apoptosis of DN mice. Therefore, we speculated that miR-203-3p might be related to the development of DN, but our study does not provide any evidence. In animal experiments, diabetic mice (db/db) were transfected with iR-203-3p overexpression lentiviral vectors (LV-miR-203-3p) and their control (LV-miR-con), with normal mice (db/m) being used as the control. High glucose (HG)-induced podocytes were used to construct a DN cell model *in vitro*. The expression levels of miR-203-3p, Semaphorin 3A (Sema3A) and inflammatory cytokines were detected by quantitative real-time polymerase chain reaction. Also, serum creatinine and blood urea nitrogen levels were used to evaluate the degree of renal injury in DN mice. Sema3A and apoptosis-related protein levels were assessed by the western blot analysis. Enzyme-linked immunosorbent assay was used to determine the different oxidative stress-related indicators and inflammatory cytokines. Flow cytometry and caspase-3 activity detection were used to analyze the degree of podocyte apoptosis. Our results suggested that the expression of miR-203-3p was lower in DN mice and in HG-induced podocytes. Overexpression of miR-203-3p reduced the body weight, blood glucose and renal injury of DN mice *in vivo*, as well as relieve the oxidative stress, inflammatory response and apoptosis of HG-induced podocytes *in vitro*. Functionally, Sema3A was a target of miR-203-3p, and Sema3A overexpression reversed the inhibitory effect of miR-203-3p on HG-induced podocyte injury. Our findings revealed that miR-203-3p alleviated the podocyte injury induced by HG via regulating Sema3A expression, suggesting that miR-203-3p might be a new therapeutic target to improve the progression of DN.

## 1 Introduction

Diabetic nephropathy (DN) is one of the most severe and harmful chronic complications of diabetes, which can cause end-stage renal disease [1,2]. According to research findings, renal disease is the leading cause of death and disability in diabetic patients [3]. DN has become a major global public health problem, which seriously affects people’s living standards. Therefore, the study of the molecular mechanisms of DN can provide a more targeted approach for the treatment of DN.

At present, metabolic damage caused by hyperglycemia is the key to the development of DN [4,5]. High glucose (HG) can cause podocyte injury, which is an essential factor for DN progression [6]. Meanwhile, DN is accompanied by renal oxidative stress and inflammatory response [7,8]. Studies have shown that HG can induce the apoptosis, oxidative stress and inflammatory response of podocytes [9,10,11]. Therefore, exploring the molecular mechanism of inhibiting podocyte injury caused by HG is helpful in developing new strategies for treating DN.

MicroRNAs (miRNAs) are single-stranded non-coding RNAs of about 22 nucleotides in length, which are involved in the regulation of gene expression post-transcription [12,13]. Substantial evidence has indicated that miRNAs might participate in the pathogenesis of DN, acting as biomarkers for DN therapy [14,15], including miR-25, miR-27b-3p and miR-1228-3p [16,17]. MiR-203-3p plays a role in the development of many diseases. For example, the downregulation of miR-203-3p promotes interleukin-33 and accelerates the course of disease in schistosomiasis infection [18]. In addition, miR-203-3p was believed to aggravate seizure activity [19], and increased miR-203-3p has been shown to inhibit the development of esophageal cancer [20]. Ji et al. suggested that long non-coding RNA Gm6135 could sponge miR-203-3p to promote the proliferation and inhibit the apoptosis of mesangial cells in DN mice, which indicated that miR-203-3p might play a negative regulatory role in the development of DN [21]. However, the current study is inconclusive.

This study aimed to explore the effect and mechanism of miR-203-3p on the oxidative stress, inflammatory response and apoptosis of podocytes induced by HG to provide potential therapeutic targets for DN.

## 2 Materials and methods

### 2.1 Animal experiments

One-month-old male diabetic mice (db/db) and control normal mice (db/m) were bought from the Model Animal Research Center of Nanjing University (Nanjing, China). After 2 weeks of adaptive feeding, db/db mice were randomly divided into three groups (*n* = 5 for every group). MiR-203-3p overexpression lentiviral vectors and their control (LV-miR-203-3p and LV-miR-con) were obtained from Genechem (Shanghai, China) and then dissolved in saline solution. Then, 4.5 nmol of LV-miR-203-3p and LV-miR-con was injected into mice by caudal vein every 3 days for a total of 30 days. After that, mice blood was collected from the caudal vein, and the blood glucose was measured by a glucometer (Yuwell, Jiangsu, China). Serum creatinine (Scr) and blood urea nitrogen (BUN) levels were measured using the corresponding Assay Kits (Amyjet Scientific, Wuhan, China). Renal tissue samples were taken from mice to detect the miR-203-3p expression.


**Ethical approval:** The research related to animal use has been complied with all the relevant national regulations and institutional policies for the care and use of animals and has been approved by the Animal Ethical Committee of The Third People’s Hospital of Dongguan City.

### 2.2 Podocyte culture and transfection

Mice podocytes were obtained from BeNa Culture Collection (BNCC, Suzhou, China) and cultured in RPMI-1640 medium (Gibco, Grand Island, NY, USA) containing 10% fetal bovine serum (Gibco), 100 U/mL penicillin and 0.1 mg/mL streptomycin at 37°C in 5% CO_2_. Meanwhile, different concentrations (0, 10, 20, 30 and 40 mM) of HG were used to treat the podocytes at different times (0, 12, 24 and 48 h) to determine the gene expression pattern and confirm the optimal concentration and time. A miR-203-3p mimic and inhibitor (miR-203-3p and anti-miR-203-3p) or their negative controls (miR-con and anti-miR-con), as well as a Semaphorin 3A (Sema3A) overexpression plasmid and its negative control (pcDNA) were synthesized by GenePharma (Shanghai, China). All plasmid oligonucleotides were transfected into podocytes using Lipofectamine 3000 (Invitrogen, Carlsbad, CA, USA).

### 2.3 Quantitative real-time polymerase chain reaction (qRT-PCR)

Total RNA was extracted by the Trizol reagent (Takara, Dalian, China) and reverse transcribed using the PrimeScript™ RT Master Mix (Takara). qRT-PCR was carried out using SYBR Green (Takara) on an ABI 7500 real-time PCR system (Applied Biosystems, Foster City, CA, USA). U6 and β-actin were used as internal controls. The primer sequences used were as follows: miR-203-3p: F 5′-GGCGGGCTGAAATGTTTAGGA-3′, R 5′-GTGCAGGGTCCGAGGTATTC-3′; U6: F 5′-CTCGCTTCGGCAGCACATATACT-3′, R 5′-CGCTTCACGAATTTGCGTGT-3′; Sema3A: F 5′-CCTCGCTCGGGACCCTT-3′, R 5′-CTTTGCAGTAGGAAAATAGCGTGA-3′; tumor necrosis factor-α (TNF-α): F 5′-CCGGAGAGGAGACTTCACAG-3′, R 5′-ACAGTGCATCATCGCTGTTC-3′; interleukin-1β (IL-1β): F 5′-CCATTACCAGCGACCCCACAG-3′, R 5′-GGGCACGTGGGCGGTATCT-3′; interleukin-6 (IL-6): 5′-CCAAAGCCTGAGCCCAGA-3′, R 5′-GCACCACTCCCATGGCAT-3′; and β-actin: F 5′-ATGATGATATCGCCGCGCTC-3′, R 5′-CCACCATCACGCCCTGG-3′. The relative gene expression was calculated using 2^−ΔΔCt^ methods. The same experiment was repeated three times, and the average was calculated.

### 2.4 Dual-luciferase reporter assay

The 3′-UTR of Sema3A containing the miR-203-3p binding sites or mutant binding sites was inserted into the pGL3-control vectors (Promega, Madison, WI, USA) to build the wild-type and mutant-type Sema3A (Sema3A-WT and Sema3A-MUT) reporter vectors. The aforementioned reporter vectors were co-transfected with different concentrations of miR-203-3p mimic (25, 50 and 100 nM) or miR-con into HG-induced podocytes. Luciferase activities were detected by the Dual-Luciferase Reporter Assay System (Promega). The same experiment was repeated three times, and the average was calculated.

### 2.5 RNA immunoprecipitation (RIP) assay

After transfection with miR-203-3p mimic or miR-con, podocytes were lysed using lysis buffer (Beyotime, Shanghai, China). After centrifugation, the podocyte supernatant was collected into a new centrifuge tube and then incubated with magnetic beads and immunoglobulin G (IgG) or Argonaute2 (Ago2) immunoprecipitation complex (Sigma-Aldrich, St. Louis, MO, USA) overnight at 4°C. Then, the beads were washed to extract RNA for qRT-PCR analysis. The same experiment was repeated three times, and the average was calculated.

### 2.6 Western blot (WB) analysis

Podocytes were lysed using lysis buffer (Beyotime). Total proteins were separated on 10% sodium dodecyl sulfate-polyacrylamide gel electrophoresis gel and then transferred onto polyvinylidene fluoride membranes (Millipore, Billerica, MA, USA). After blockage with non-fat milk, membranes were incubated with the primary antibodies against Sema3A (1:400, PA5-77752; Invitrogen), B-cell lymphoma-2 (Bcl-2; 1:50, MA5-11757; Invitrogen), Bcl-2-associated X (Bax; 1:100, MA5-14003; Invitrogen) or β-actin (1:1,500, MA5-15739; Invitrogen) overnight at 4°C. Subsequently, the incubation with the secondary antibody (1:2,000; Invitrogen) was performed for 1 h. Band detection was exposed via enhanced chemiluminescence solution (Beyotime). The same experiment was repeated three times, and the average was calculated.

### 2.7 Enzyme-linked immunosorbent assay (ELISA)

Podocytes were collected into the centrifuge tube and repeatedly defrosted and frozen for 3–4 times until the cells were completely broken. The supernatant of podocytes was collected after centrifugation for ELISA. Reactive oxygen species (ROS) formation, malondialdehyde (MDA) level and superoxide dismutase (SOD) activity were used to evaluate the degree of oxidative stress in podocytes using the corresponding ELISA Kits (Nanjing Jiancheng Bioengineering Institute, Nanjing, China). The complement of TNF-α, IL-1β and IL-6 was used to measure the degree of inflammatory response in podocytes using the corresponding ELISA Kits (Goibibo, Shanghai, China). The same experiment was repeated three times, and the average was calculated.

### 2.8 Apoptotic analysis and caspase-3 activity detection

After treatment or transfection, podocytes were digested with trypsin and collected into the centrifuge tube. Thoroughly centrifuged and washed, podocytes were resuspended with binding buffer (BD Biosciences, Franklin Lakes, NJ, USA) and incubated with FITC Annexin V + propidium iodide reagents (BD Biosciences) for 15 min to detect the apoptotic rate using flow cytometry (Merck KGaA, Darmstadt, Germany). Moreover, podocytes were resuspended with lysis buffer (Beyotime) and lysed for 15 min. The supernatant was collected to measure the activity of caspase-3 using Caspase-3 Activity Detection Kit (Beyotime). The same experiment was repeated three times, and the average was calculated.

### 2.9 Statistical analysis

Results were presented as mean ± standard deviation. Statistical analysis was carried out using Student’s *t*-test or one-way analysis of variance. Data analysis was performed using GraphPad Prism 7.0 (GraphPad, San Jose, CA, USA). A *P* value of <0.05 was considered to be statistically significant.

## 3 Results

### 3.1 MiR-203-3p expression was lower in db/db mice and its overexpression alleviated renal injury

**Figure 1:**
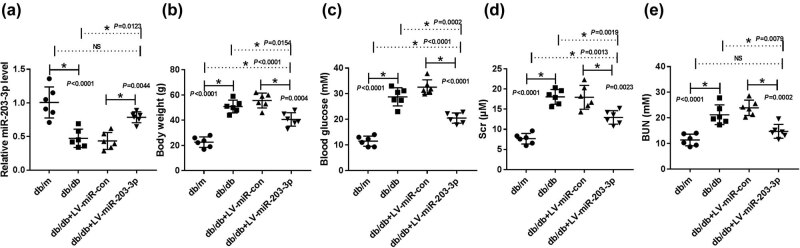
Effects of miR-203-3p expression on the biochemical indicators in DN mice. (a) The level of miR-203-3p in db/db mice was detected by qRT-PCR. (b) Body weight was measured in mice. (c) Blood glucose was determined by a glucometer. (d and e) The concentration of Scr and BUN was measured by the corresponding assay kits. *P < 0.05.

To investigate the potential role of miR-203-3p in DN, we first examined the expression of miR-203-3p in db/db mice. qRT-PCR results showed that compared with db/m mice, miR-203-3p expression was significantly downregulated in db/db mice and was markedly increased after LV-miR-203-3p transfection (*P* < 0.05, Figure 1a). The monitoring of body weight showed that high body weight of db/db mice was reduced by the aberrant miR-203-3p expression (*P* < 0.05, Figure 1b). Also, similar results were found for blood glucose, Scr and BUN levels of mice. As shown in Figure 1c–e, overexpression of miR-203-3p decreased the blood glucose, Src and BUN levels in db/db mice (*P* < 0.01), indicating that miR-203-3p overexpression ameliorated the renal injury of db/db mice. These results confirmed that miR-203-3p played a vital role in DN.

### 3.2 MiR-203-3p expression was decreased in HG-induced podocytes

**Figure 2:**
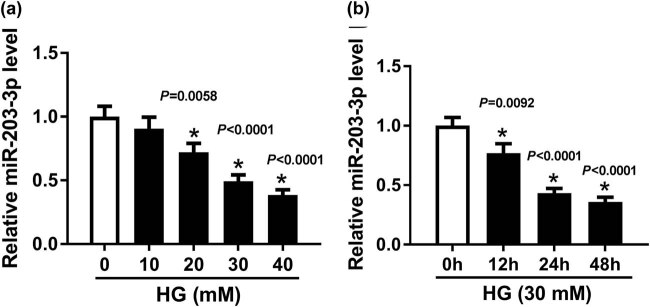
MiR-203-3p was decreased in podocytes exposed to HG. (a) The expression of miR-203-3p in podocytes treated with different concentrations of HG (0, 10, 20, 30 and 40 mM) was measured by qRT-PCR. (b) qRT-PCR was conducted to analyze the expression of miR-203-3p in podocytes treated with 30 mM HG at specified time points (0, 12, 24 and 48 h). *P < 0.05.

At the same time, we investigated the expression of miR-203-3p in HG-induced podocytes. qRT-PCR was performed to detect the expression of miR-203-3p in different concentrations of glucose (0, 10, 20, 30 and 40 mM) and different times (0, 12, 24 and 48 h) after HG treatment. As shown in Figure 2a and b, the expression of miR-203-3p was decreased in HG-induced podocytes in a concentration-dependent manner and time-dependent manner (*P* < 0.01). When the concentration of HG was 30 mM, the inhibition effect on the miR-203-3p level reached 50%. Therefore, subsequent experiments were conducted using HG with a concentration of 30 mM for 48 h.

### 3.3 Sema3A was a target of miR-203-3p

**Figure 3:**
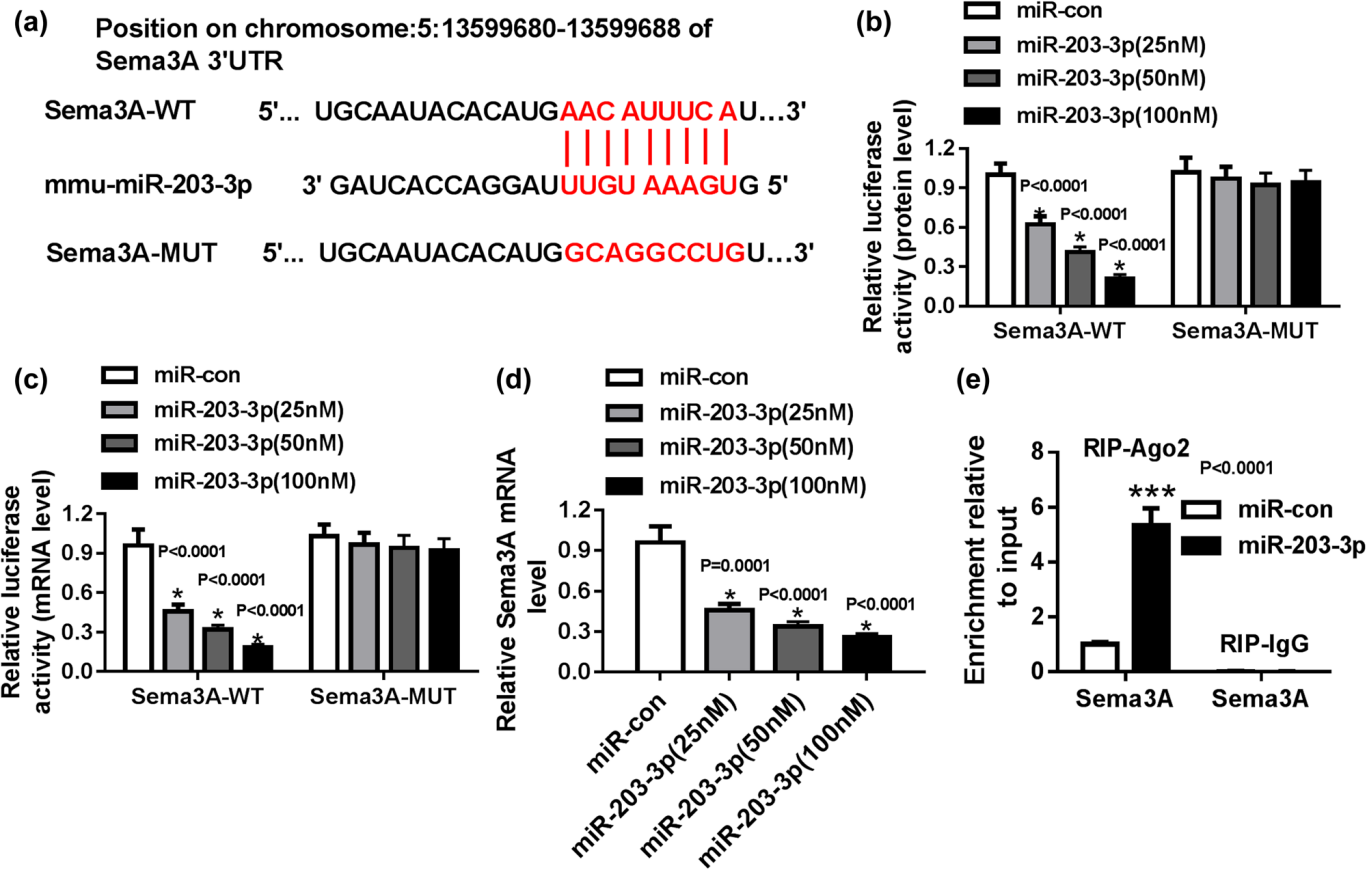
MiR-203-3p targeted Sema3A. (a) The predicted binding sites and mutant binding sites between Sema3A 3′-UTR and miR-203-3p are shown. (b and c) Luciferase activities (protein and mRNA levels) were detected after co-transfection with Sema3A-WT or Sema3A-MUT reporter vectors and miR-con or miR-203-3p mimic. (d) After transfection with different concentrations of miR-203-3p mimic into HG-induced podocytes, the mRNA level of Sema3A was detected by qRT-PCR. (e) RIP assay was conducted to examine the Sema3A enrichment pattern in IgG or Ago2 immunoprecipitation complex (RIP-IgG or RIP-Ago2). *P < 0.05, ***P < 0.001.

We used the microT-CDS tool to explore the downstream target gene of miR-203-3p and predicted that Sema3A 3′-UTR had complementary binding sites with miR-203-3p (Figure 3a). According to the binding sequence, we constructed the Sema3A-WT and Sema3A-MUT reporter vectors for dual-luciferase reporter assay. We found that under the treatment of different concentrations of miR-203-3p, the luciferase activity (protein and mRNA levels) of the Sema3A-WT reporter vector was significantly decreased in a dose-dependent manner (*P* < 0.0001), while the luciferase activity of the Sema3A-MUT reporter vector had not changed (Figure 3b and c). As well, we also discovered that the expression of Sema3A was markedly reduced with the increase of miR-203-3p concentration (*P* < 0.001, Figure 3d). Furthermore, the RIP assay further revealed that the ectopic expression of miR-203-3p resulted in the abundant enrichment of Sema3A in RIP-Ago2 (Figure 3e).

### 3.4 Sema3A was highly expressed in HG-induced podocytes and its expression was regulated by miR-203-3p

**Figure 4:**
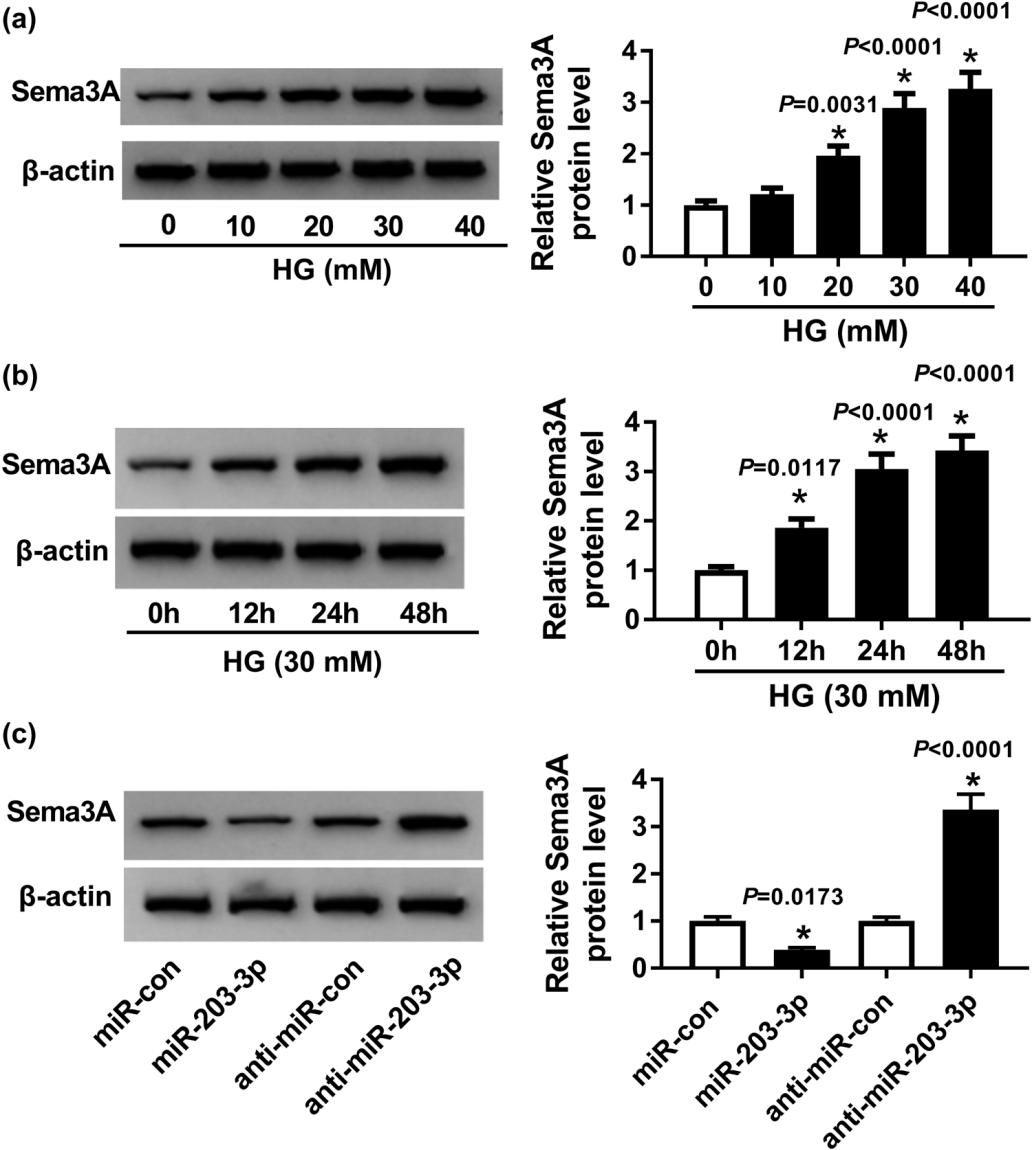
Sema3A expression was increased in podocytes exposed to HG. (a and b) The WB analysis was performed to assess the protein level of Sema3A in different concentrations of glucose (0, 10, 20, 30 and 40 mM) or time points (0, 12, 24 and 48 h) of HG treatment. (c) The effect of miR-203-3p expression on Sema3A protein level was detected by the WB analysis. *P < 0.05.

Meanwhile, we measured the Sema3A expression in HG-induced podocytes. WB results indicated that the protein level of Sema3A was increased with increasing HG concentration and treatment time (*P* < 0.05, Figure 4a and b). Moreover, we also found that miR-203-3p overexpression markedly inhibited the protein level of Sema3A, while miR-203-3p inhibition enhanced its protein level (*P* < 0.05, Figure 4c). These results confirmed that miR-203-3p targeted Sema3A in HG-induced podocytes.

### 3.5 Overexpressed Sema3A reversed the inhibition effect of miR-203-3p on the oxidative stress in HG-induced podocytes

**Figure 5:**
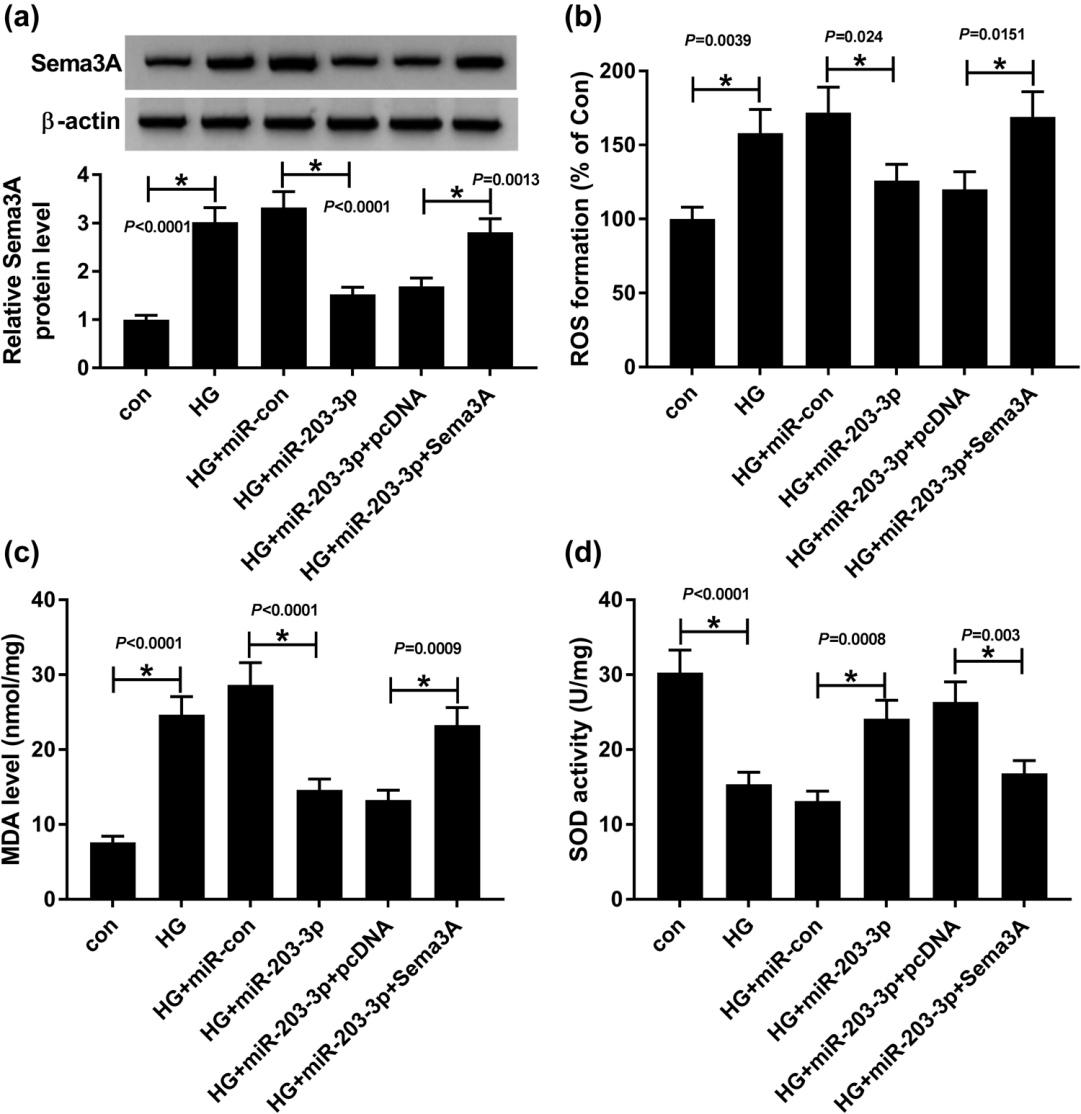
MiR-203-3p and Sema3A regulated the oxidative stress of HG-induced podocytes. The HG-induced podocytes were transfected with miR-con, miR-203-3p, miR-203-3p + pcDNA or miR-203-3p + Sema3A. (a) The protein level of Sema3A was determined by the WB analysis. (b–d) ROS production, MDA level and SOD activity were measured by the corresponding ELISA kits. *P < 0.05.

To investigate the impacts of miR-203-3p and Sema3A on HG-induced podocytes, we transfected HG-induced podocytes with miR-203-3p mimic and Sema3A overexpression plasmids to detect related effects on the physiological indicators in podocytes. The WB analysis showed that the Sema3A expression was significantly increased in HG-induced podocytes, while miR-203-3p overexpression reduced the Sema3A expression (*P* < 0.01, Figure 5a). As shown in Figure 5b–d, the stimulation of HG led to the accumulation of ROS and MDA levels and the inhibition of SOD activity, indicating that HG can induce the oxidative stress in podocytes (*P* < 0.01). Overexpression of miR-203-3p effectively reduced ROS and MDA levels and enhanced the SOD activity (*P* < 0.05), while Sema3A overexpression blocked the inhibition effects of miR-203-3p on oxidative stress (*P* < 0.05). These results strongly suggested that miR-203-3p had hindering effects on the oxidative stress of HG-induced podocytes through inhibiting Sema3A expression.

### 3.6 Sema3A overexpression reversed the suppression effect of miR-203-3p on the inflammatory response of HG-induced podocytes

**Figure 6:**
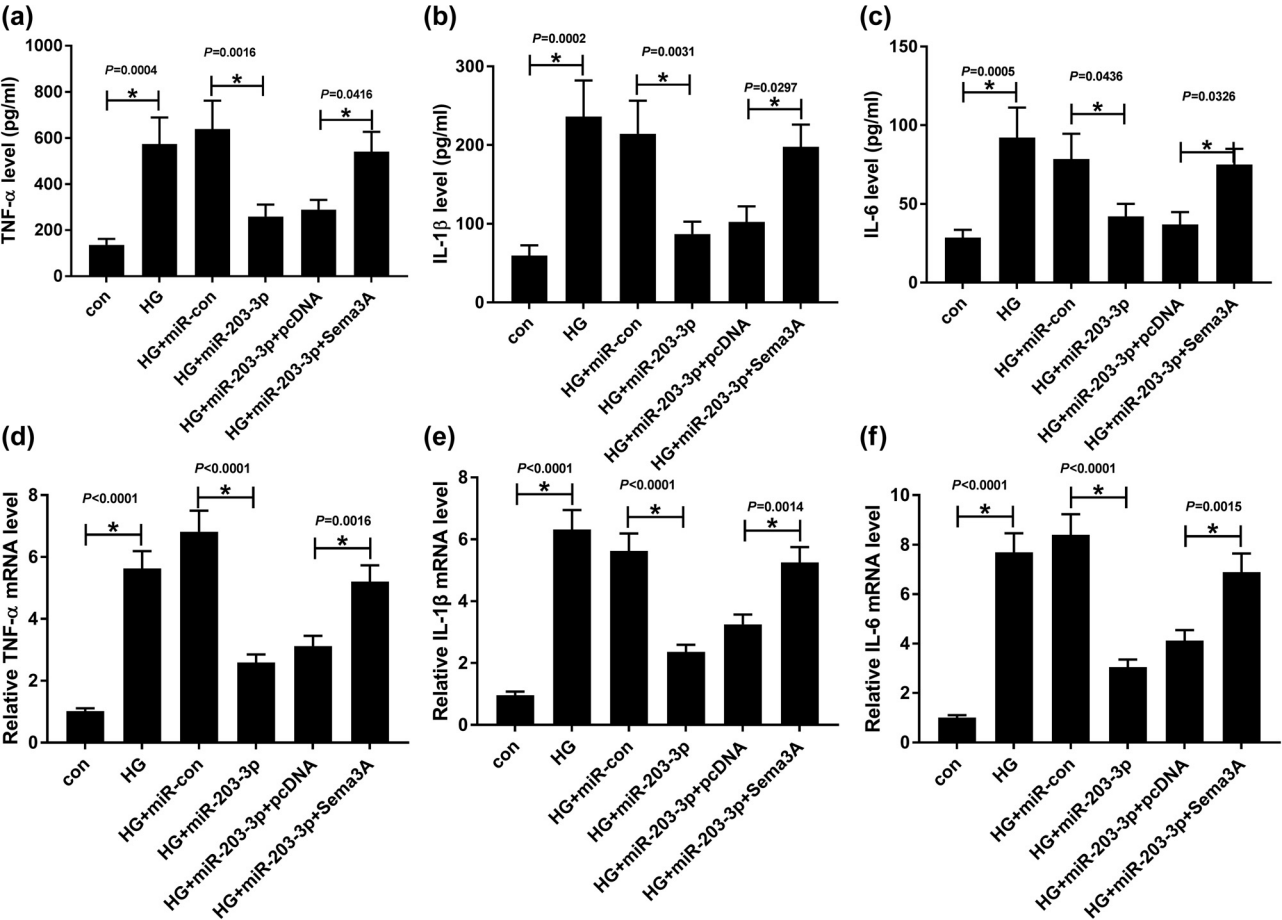
MiR-203-3p and Sema3A regulated the inflammatory response of HG-induced podocytes. HG-induced podocytes were transfected with miR-con, miR-203-3p, miR-203-3p + pcDNA or miR-203-3p + Sema3A. (a–c) ELISA was performed to measure the concentrations of inflammatory cytokines TNF-α, IL-1β and IL-6. (d–f) The mRNA levels of these inflammatory cytokines were detected by qRT-PCR. * *P* < 0.05.

Subsequently, we investigated the impact of miR-203-3p and Sema3A on the inflammatory response of podocytes. Through detecting the complement of inflammatory cytokines by ELISA and qRT-PCR, we found that the overexpression of miR-203-3p could inhibit the stimulation effects of HG on the levels of TNF-α, IL-1β and IL-6 in podocytes, while the Sema3A overexpression could reverse the inhibition effect of miR-203-3p on the levels of TNF-α, IL-1β and IL-6 (*P* < 0.05, Figure 6a–f). All data revealed that miR-203-3p suppressed the Sema3A expression to restrain the inflammatory response of HG-induced podocytes.

### 3.7 Upregulation of Sema3A reversed the inhibitory effect of miR-203-3p on the apoptosis of HG-induced podocytes

**Figure 7:**
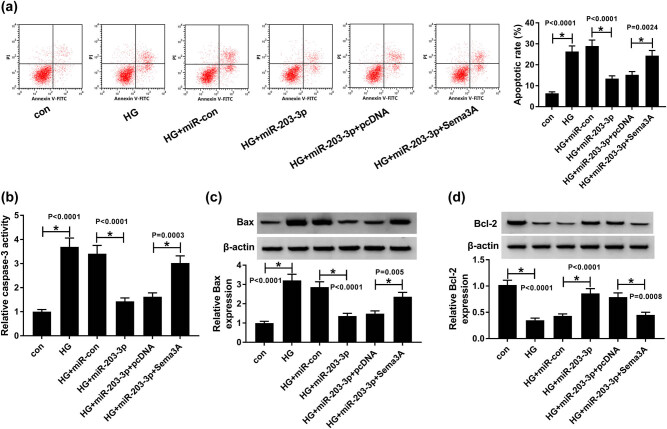
MiR-203-3p and Sema3A regulated the apoptosis of HG-induced podocytes. HG-induced podocytes were transfected with miR-con, miR-203-3p, miR-203-3p + pcDNA or miR-203-3p + Sema3A. (a) Podocyte apoptosis was evaluated using flow cytometry. (b) The activity of caspase-3 was measured by the Caspase-3 Activity Detection Kit. (c and d) The WB analysis determined the protein levels of apoptosis-associated markers Bax and Bcl-2. *P < 0.05.

Furthermore, flow cytometry results showed that the ectopic expression of miR-203-3p significantly reduced the podocytes apoptosis induced by HG, while the addition of the Sema3A overexpression plasmid inverted this effect (*P* < 0.01, Figure 7a). Also, the caspase-3 activity detection results suggested that miR-203-3p overexpression hindered the activity of caspase-3, while Sema3A increased its activity (*P* < 0.001, Figure 7b). The WB results indicated that overexpressed miR-203-3p promoted the Bax protein level and inhibited the Bcl-2 protein level. Instead, the overexpression of Sema3A reversed the function of miR-203-3p (*P* < 0.01, Figure 7c and d). These results confirmed that miR-203-3p blocked the podocyte apoptosis induced by HG through reducing Sema3A expression.

## 4 Discussion

MiRNAs are associated with various cytological processes in DN, including podocyte injury, insulin resistance and inflammatory response [22,23,24]. In this study, we found that the miR-203-3p expression was markedly decreased in the renal tissue of db/db mice. After transfection of miR-203-3p overexpression lentivirus vectors, the high body weight, high blood sugar and renal injury of db/db mice were all significantly alleviated. Thus, this finding indicated that miR-203-3p might play a role in reversing or preventing the development and progression of DN.

HG-induced podocyte injury is the key to establishing the DN model *in vitro* [25,26]. We detected the miR-203-3p expression in HG-induced podocytes and found that the miR-203-3p level was reduced in a concentration-dependent manner and time-dependent manner, indicating that miR-203-3p might be involved in HG-induced podocytes injury. Subsequently, through the target gene prediction and analysis, we determined that Sema3A could interact with miR-203-3p. Sema3A, a member of the semaphorin family, was initially discovered to be involved in the development of neurons [27]. The results of Aggarwal et al. indicated that the overexpression of Sema3A accelerated advanced DN and might act as a novel potential therapeutic target for DN [28]. Also, Sema3A could be used as a target gene of miR-15b-5p to participate in the regulation of HG-induced podocytes by miR-15b-5p [29]. Here, we also found that the Sema3A level was increased in HG-induced podocytes and was regulated by the miR-203-3p expression.

**Figure A1:**
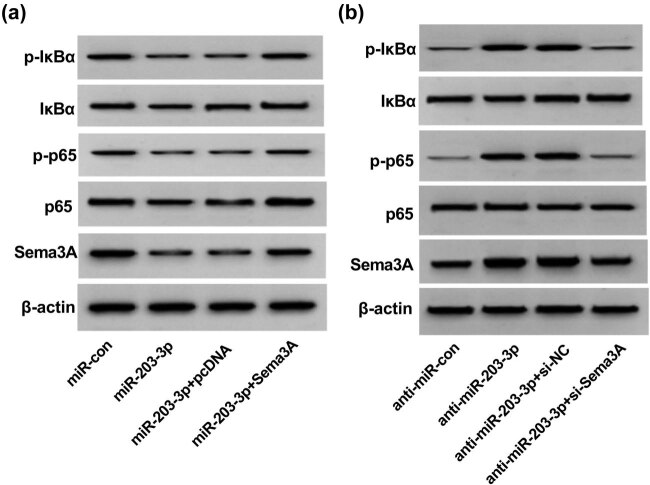
The expression of NF/κB signaling pathway-related markers. (a) HG-induced podocytes were transfected with miR-con, miR-203-3p, miR-203-3p + pcDNA or miR-203-3p + Sema3A. The WB analysis was used to measure the protein expression of p-IκBα, IκBα, p-p65 and p65. (b) HG-induced podocytes were transfected with anti-miR-con, anti-miR-203-3p, miR-203-3p + si-NC or miR-203-3p + si-Sema3A. The protein expression levels of p-IκBα, IκBα, p-p65 and p65 were detected by the WB analysis.

Oxidative stress and inflammatory response are two major factors promoting the development of DN [30,31]. Evidence has suggested that podocyte apoptosis has played a key role in the pathophysiology of DN [32,33]. Therefore, the detection of oxidative stress, inflammatory response and apoptosis-related indicators of podocytes could reflect the degree of damage of podocytes. In this study, we discovered that the miR-203-3p overexpression could reduce the oxidative stress, inflammatory response and apoptosis of podocytes induced by HG. Meanwhile, overexpression of Sema3A reversed the inhibitory effects of miR-203-3p to promote podocyte injury. These results confirmed that miR-203-3p inhibited podocyte injury by regulating the expression of Sema3A. However, the specific mechanism of miR-203-3p in regulating the inflammation and oxidative stress has not been thoroughly studied. NF/κB is a classical signaling pathway involved in cellular inflammation and oxidative stress. To investigate whether the miR-203-3p/Sema3A axis mediated the activity of the NF/κB signaling pathway in regulating the HG-induced podocyte injury, we detected the expression of the NF/κB signaling pathway-related markers in HG-induced podocytes co-transfected with miR-203-3p and Sema3A, as well as with anti-miR-203-3p and si-Sema3A. As shown in Figure A1, we found that the overexpression of miR-203-3p significantly inhibited the protein levels of p-IκBα and p-p65, and this inhibition could be reversed by the overexpression of Sema3A. In addition, anti-miR-203-3p could also significantly promote the protein levels of p-IκBα and p-p65, and the addition of si-Sema3A also reversed this promoting effect. Therefore, we speculated that miR-203-3p mediated the inflammation and oxidative stress by regulating the activity of the NF/κB signaling pathway through Sema3A, which needs to be confirmed by further experiments.

In summary, we provided the conclusion that miR-203-3p alleviated oxidative stress, inflammatory response and apoptosis in podocytes by targeting Sema3A to improve HG-induced podocyte injury. Hence, these findings provide novel insights into the beneficial role of miR-203-3p in DN. However, the current study has some limitations. We explored only the regulation of miR-203-3p-mediated HG-induced podocyte injury by targeting Sema3A in DN cell models *in vitro*, but this aspect had not been further confirmed *in vivo*. In future studies, we will conduct *in vivo* experiments to further explore this point.
